# Supplementary material for: Heat adaptation of phage T7 under an extended genetic code
Source: Virus Evol. 2021 Dec 1;7(2):veab100. doi: 10.1093/ve/veab100 (PMC8923235; doi:10.1093/ve/veab100)
Supplement: veab100_Supp [file veab100_supp.zip › Sequencing_Analysis.zip!/Sequencing_Analysis/S2/evidence/JC_SIDE_2_65.html]

BRESEQ :: Evidence


| New junction evidence | | | | | | | | | | | |
| --- | --- | --- | --- | --- | --- | --- | --- | --- | --- | --- | --- |
|  | | seq id | position | reads (cov) | reads (cov) | score | skew | freq | annotation | gene | product |
| \* | ? | NC\_001604.1 | = 13586 | 0 (0.000) | 46 (0.010) | 20/592 | 45.7 | 50.3% | intergenic (–/–) | *–/–* | –/– |
|  | ? | NC\_001604.1 | 14356 = | 91 (0.020) |  |  |  |  | intergenic (–/–) | *–/–* | –/– |
| Rejected: Coverage evenness skew score above cutoff. | | | | | | | | | | | |

Only 100 of 5460 total aligned reads displayed.  

`GACAGACTGGCTGGAAGAAACCAGAAGTTACCAAGCTGAGTCATAAGGTGTTCGATAAGGACACTATGACCCACATCAAGGCTGGTGATTGGGTTAAGGTTGACATGGGAGTTGTTGGTGGATACGGCTACGTCCGCTCAGTTAGTGGCAAATATGCACAAGTGTCATACATCACAGGTGTTACTCCACGCGGTGCAATCGTTGCCGATAAGACCAACATGATTCACACAGGTTTCTTGACAGTTGTTTCATATGAAGAGATTGTTAAGTCACGATAATCAATAGGAGAAATCAATATGATCGTTTCTGACATCGAAGCTAACGCCCTCTTAGAGAGCGTCACTAAGTTCCACTGCGGGGTTATCTACGACTACTCCACCGCTGAGTACGTAAGCTACCGTCCGAGTGACTTCGGTGCGTATCTGGATGCGCTGGAAGCCGAGGTTGCACGAGGCGGTCTTATTGTGTTCCACAACGGTCACAAGTATGACGTTCCTGCATTGACCAAACTGGCAAAGTTGCAATTGAACCGAGAGTTCCACCTTCCTCGTGAGAACTGTATTGACACCCTTGTGTTGTCACGTTTGATTCATTCCAACC  >  NC_001604.1/14057‑14656`  

                                                                                                                                                                                                                                                                                                           `|`                                                                                                                                                                                                                                                                                                              
`gacaagtacgagaagtttcgatggagagtcccattctaatacgactcactaaaggagacacaccatgttcaaactgattaagaagttaggccaactgctggttcgtatgtacaacgtggaagccaagcgactgaacgatgaggctcgtaaagaggccacacagtcacgcgctctggcgattctctccaacgaactggctgacagtgcatccactaaagttaccgaggctgcccgtgtggcaaaccaagctcaacagctttccaaattctttgagtaatcaaacaggagaaaTCAATATGAt                                                                                                                                                                                                                                                                                                             <  1:643200‑M2/10‑1 (MQ=255)`  
            `aagtttcgctggagagtcccattctaatacgactcactaaaggagacacaccatgttcaaactgattaagaagttaggccaactgctggctcgtatgtacaacgtggaagccaagcgactgaacgatgaggctcgtaaagaggccacacagtcacgcgctctggcgattcgctccaacgaactggctgacagtgcatccacaaaagttaccgaggctgcccgtgtggcaaaccaagctcaacagctttccaaattctttgagtaatcaaacaggagaaaTCAATATGATCGTTTCTGACAt                                                                                                                                                                                                                                                                                                 <  1:411599‑M2/22‑1 (MQ=255)`  
                      `ggagagtcccattctaatacgactcactaaaggagacacaccatgttcaaactgattaagaagttaggccaactgctggttcgtatgtacaacgtggaagccaagcgactgaacgatgaggctcgtaaagaggccacacagtcacgcgctctggcgattcgctccaacgaactggctgacagtgcatccactaaagttaccgaggctgcccgtgtggcaaaccaagctcaacagctttccaaattctttgagtaatcaaacaggagaaaTCAATATgatcgtttctgacctgtctcttata                                                                                                                                                                                                                                                                                       >  1:534303‑M2/270‑289 (MQ=255)`  
                           `gtcccattctaatacgactcactaaaggagacacaccatgttcaaactgattaagaagttaggccaactgctggttcgtatgtacaacgtggaagccaagcgactgaacgatgaggctcgtaaagaggccacacagtcacgcgctctggcgattcgctccaacgaactggctgacagtgcatccactaaagttaccgaggctgcccgtttggcaaaccaagctcaacagctttccaaattctttgagtaatcaaacaggagaaaTCAATATGATCGTTTCTGACATCGAAGCTAACGCCct                                                                                                                                                                                                                                                                                  >  1:288041‑M2/265‑301 (MQ=255)`  
                           `gtcccattctaatacgactcactaaaggagacacaccatgttcaaactgattaagaagttaggccaactgctggttcgtatgtacaacgtggaagccaagcgactgaacgatgaggctcgtaaagaggccacacagtcacgcgctctggcgattcgctccaacgaactggctgacagtgcatccactaaagttaccgaggatgcccgtgtggcaaaccaagctcaacagctttccaaattctttgagtaatcaaacaggagaaaCCATTATGATCGTTTCTGACATCGAAGCTAACGCCct                                                                                                                                                                                                                                                                                  >  1:100118‑M2/265‑301 (MQ=255)`  
                                `attctaatacgactcactaaaggagacacaccatgttcaaactgattaagaagttaggccaactgctggttcgtatgtacaacgtggaagccaagcgactgaacgatgaggctcgtaaagaggccacacagtcacgtgctctggcgattcgctccaacgaactggctgacagtgcatccactaaagttaccgaggctgcccgtgtggcaaaccaagctcaacagctttccaaattctttgagtaatcaaacaggagaaaCCATTATGATCGTTTCTGACATCGAAGCTAACGCCCTCTTag                                                                                                                                                                                                                                                                             <  1:55569‑M2/42‑1 (MQ=255)`  
                                 `ttctaatacgactcactaaaggagacacaccatgttcaaactgattaagaagttaggccaactgctggttcgtatgtacaacgtggaagccaagcgactgaacgatgaggctcgtaaagaggccacacagtcacgcgctctggcgattcgctccaacgaactggctgacagtgcatccactaaagttaccgaggctgcccgtgtgtcaaaccaagctcaacagctttccaaattctttgagtaatcaaacaggagaaaTCAATATGATCGTTTCTGACATCGAAGCTAACGCCCTCTTaga                                                                                                                                                                                                                                                                            >  1:61532‑M2/259‑301 (MQ=255)`  
                                 `ttctaatacgactcactaaaggagacacaccatgttcaaactgattaagaagttaggccaactgctggttcgtatgtacaacgtggaagccaagcgactgaacgatgaggctcgtaaagaggccacacagtcacgcgctctggcgattcgctccaacgaactggctgacagtgcatccactaaagttaccgaggctgcccgtgtggcaaaccaagctcaacagctttccaaattctttgagtaatcaaacaggagaaaTCAATATGATCGTTTCTGACATCGAAGCTAACGCCCTCTTaga                                                                                                                                                                                                                                                                            >  1:799634‑M2/259‑301 (MQ=255)`  
                                 `ttctaatacgactcactaaaggagacacaccatgttcaaactgattaagaagttaggccaactgctggttcgtatgtacaacgtggaagccaagcgactgaacgatgaggctcgtaaagaggccacacagtcacgcgctctggcgattcgctccaacgaactggctgacagtgcatccactaaagttaccgaggctgcccgtgtggcaaaccaagctcaacagctttccaaattctttgagtaatcaaacaggagaaaTCAATATGATCGTTTCTGACATCGAAGCTAACGCCCTCTTaga                                                                                                                                                                                                                                                                            >  1:742472‑M2/259‑301 (MQ=255)`  
                                 `ttctaatacgactcactaaaggagacacaccatgttcaaactgattaagaagttaggccaactgctggttcgtatgtacaacgtggaagccaagcgactgaacgatgaggctcgtaaagaggccacacagtcacgcgctctggcgattcgctccaacgaactggctgacagtgcatccactaaagttaccgaggctgcccgtgtggcaaaccaagctcaacagctttccaaattctctgagtaatcaaacaggggaaaTCAATATGATAGATTCTGACAGAGAAGCTAACGCCCTCTTaga                                                                                                                                                                                                                                                                            >  1:152075‑M2/259‑301 (MQ=255)`  
                                  `ttctaatacgactcactaaaggagacacaccatgttcaactgattaagaagttaggccaactgctggttcgtatgtacaacgtggaagccaagcgactgaacgatgaggctcgtaaagaggccacacagtcacgcgctctggcgattcgctccaacgaactggctgacagtgcatccactaaagttaccgaggctgcccgtgtggcaaaccaagctcaacagctttccaaattctttgagtaatcaaacaggagaaaTCAATATGATCGTTTCTGACATCGAAGCTAACGCCCTCTTagag                                                                                                                                                                                                                                                                           <  1:660836‑M2/44‑1 (MQ=255)`  
                                  `tctactacgacacactaaaggagacacaccatgttcaaactgattaagaagttaggccaactgctggttcgtatgtacaacgtggaagccaagcgactgaacgatgaggctcgtaaagaggccacacagtcacgcgctctggcgattcgctccaacgaactggctgacagtgcatccactaaagttaccgaggctgcccgtgtggcaaaccaagctcaacagctttccaaattctttgagtaatcaaacaggagaaaTCAATATGATCGTTTCTGACATCGAAGCTAACGCCCTCTTagag                                                                                                                                                                                                                                                                           <  1:235590‑M2/44‑1 (MQ=255)`  
                                  `tctaatacgactcactaaaggagacacaccatgttcaaactgattaagaagttaggccaactgctggttcgtatgtacaacgtggaagccaagcgactgaacgatgaggctcgttaagaggccacacagtcacgcgctctggcgattcgctccaacgaactggctgacagtgcatccactaaagttaccgaggctgcccgtgtggcaaaccaagctcaacagctttccaaattctttgagtaatcaaacaggagaaaTCAATATGATCGTTTCTGACATCGAAGCTAACGCCCTCTTagag                                                                                                                                                                                                                                                                           <  1:258445‑M2/44‑1 (MQ=255)`  
                                  `tctaatacgactcactaaaggagacacaccatgttcaaactgattaagaagttaggccaactgctggttcgtatgtacaacgtggaagccaagcgactgaacgatgaggctcgtaaagaggccacacagtcacgcgctctggcgattcgctccaacgaactggctgacagtgcatccactaaagttaccgaggctgcccgtgtggcaaaccaagctcaacagctttccaaattctttgagtaatcaaacaggagaaaTCAATATGATCGTTTCTGACATCGAAGCTAACGCCCTCTTagag                                                                                                                                                                                                                                                                           <  1:165436‑M2/44‑1 (MQ=255)`  
                                  `tctaatacgactcactaaaggagacacaccatgttcaaactgattaagaagttaggccaactgctggttcgtatgtacaacgtggaagccaagcgactgaacgatgaggctcgtaaagaggccacacagtcacgcgctatggcgattcgctccaacgaactggctgacagtgcatccactaaagttaccgaggctgcccgtgtggcaaaccaagctcaacagctttccaaattctttgagtaatcaaacaggagaaaTCAATATGATAGTTTCTGACATCGAAGCTAACGCCCTCTTagag                                                                                                                                                                                                                                                                           <  1:444738‑M2/44‑1 (MQ=255)`  
                                  `tataagagacagcactaaaggagacacaccatgttcaaactgattaagaagttaggccaactgctggttcgtatgtacaacgtggaagccaagcgactgaacgatgaggctcgtaaagaggccacacagtcacgcgctctggcgattcgctccaacgaactggctgacagtgcatccactaaagttaccgaggctgcccgtgtggcaaaccaagctcaacagctttccaaattctttgagtaatcaaacaggagaaaTCAATATGATCGTTTCTGACATCGAAGCTAACGCCCTCTTagag                                                                                                                                                                                                                                                                           <  1:621089‑M2/44‑1 (MQ=255)`  
                                  `tataagagacagcactaaaggagacacaccatgttcaaactgattaagaagttaggccaactgctggttcgtatgtacaacgtggaagccaagcgactgaacgatgaggctcgtaaagaggccacacagtcacgagctctggcgattcgctccaacgaactggttgacagtgcatccactaaagttaccgaggctgcccgtgtggcaaaccaagctcaacagctttccaaattctttgagtaatcaaacaggagaaaTCAATATGATCGTTTCTGACATCGAAGCTAAGGCCCTCTTagag                                                                                                                                                                                                                                                                           <  1:337930‑M2/44‑1 (MQ=255)`  
                                  `ggagatgtgtataagagacagagacacaccatgttcaaactgattaagaagttaggccaactgctggttcgtatgtacaacgtggaagccaagcgactgaacgatgaggctcgtaaagaggccacacagtcacgcgctctggcgattcgctccaacgaactggctgacagtgcatccactaaagttaccgaggctgcccgtgtggcaaaccaagctcaacagctttccaaattctttgagtaatcaaacaggagaaaTCAATATGATCGTTTCTGACATCGAAGCTAACGCCCTCTTagag                                                                                                                                                                                                                                                                           <  1:668410‑M2/44‑1 (MQ=255)`  
                                   `ctaatacgactcactaaaggagacacaccatgttcaaactgattaagaagttaggccaactgctggttcgtatgtacaacgtggaagccaagcgactgaacgatgaggctcgtaaagaggccacacagtcacacgctctggcgattcgctccaacgaactggctgacagtgcatccactaaagttaccgaggctgcccgtgtggcaaaccaagctcaacagctttccaaattctttgagtaatcaaacaggagaaaTCAATATGATCGTTTCTGACATCGAAGCTAACGCCCTCTtagagc                                                                                                                                                                                                                                                                          >  1:505438‑M2/257‑300 (MQ=255)`  
                                    `gatgtgtataagagacaggagacacaccatgttcaaactgattaagaagttaggccaactgctggttcgtatgtacaacgtggaagccaagcgactgaacgatgaggctcgtaaagaggccacacagtcacgcgctctggcgattcgctccaacgaactggctgacagtgcatccactaaagttaccgaggctgcccgtgtggcaaaccaagctcaacagctttccaaattctttgagtaatcaaacaggagaaaTCAATATGATCGTTTCTGACATCGAAGCTAACGCCCTCTTagagag                                                                                                                                                                                                                                                                         <  1:697807‑M2/46‑1 (MQ=255)`  
                                    `aaatacgactcactaaaggagacacaccatgttcaaactgattaagaagttaggccaactgctggttcgtatgtacaacgtggaagccaagcgactgaacgatgaggctcgtaaagaggccacacagtcacgcgctctggcgattcgctccaacgaactggctgacagtgcatccactaaagttaccgaggctgtccgtgtggcaaaccaagctcaacagctttccaaattctttgagtaatcaaacaggagaaaTCAATATGATCGTTTCTGACATCGAAGCTAACGCCCTCTTagagag                                                                                                                                                                                                                                                                         <  1:83011‑M2/46‑1 (MQ=255)`  
                                            `gctcggagatgtgtataagagacagcaaactgattaagaagttaggccaactgctggttcgtatgtacaacgtggaagccaagcgactgaacgatgaggctcgtaaagaggccacacagtcacgcgctctggtgattcgctccaacgaactggctgacaatgcatccactaaagttaccgaggctgcccgtgtggcaaaccaagctcaacagctttccaaattcttggagtaatcaaacaggagaaaCCATTATGATCGTTTCTGACATCGAAGCTAACGCCCTCTTAGAGAGCGTCACTa                                                                                                                                                                                                                                                                 <  1:548953‑M2/54‑1 (MQ=255)`  
                                            `atcactaaaggagatacaccagtttcaaactgattaagaagttaggccaactgctggtgcgtatgtacaatgtggaagccaagcgactggacgatgaggcgcgtaaagaggccacacaggcactcgctctggcgattcgctccaacgaactggctgacagtgcatccactaaagttaccgaggctgccagtgtggcaaaccaagctcaacagctttctaaatgctttgagtaatcaaacaggagaaaTCAATATGATCGTTTCTGACATCGAAGCTAACGCCCTCTTAGAGAGCGTCACTa                                                                                                                                                                                                                                                                 <  1:75565‑M2/54‑1 (MQ=255)`  
                                              `cactaaaggagacacaccatgttcaaactgattaagaagttaggccaactgctggttcgtatgtacaacgtggaagccaagcgactgaacgatgaggctcgtaaagaggccacacagtcacgcgctctggcgattcgctccaacgaactggctgacagtgcatccactaaagttaccgaggctgcccgtgtggcaaaccaagctcaacagctttccaaattctttgagtaatcaaacaggagaaaTCAATATGATCGTTTCTGACATCGAAGCTAACGCCCTCTTAGAGAGCGTCACTAAg                                                                                                                                                                                                                                                               >  1:121536‑M2/246‑301 (MQ=255)`  
                                              `cactaaaggagacacaccatgttcaaactgattaagaagttaggccaactgctggttcgtatgtacaacgtggaagccaagcgactgaacgatgaggctcgtaaagaggccacacagtcacgcgctctggcgattcgctccaacgaactggctgacagtgcatccactaaagttaccgaggctgcccgtgtggcaaaccaagctcaacagctttccaaattctttgagtaatcaaacaggagaaaTCAATATGATCGTTTCTGACATCGAAGCTAACGCCCTCTTAGAGAGCGTCACTAAg                                                                                                                                                                                                                                                               >  1:251887‑M2/246‑301 (MQ=255)`  
                                               `cactaaaggagacacaccatgttcaaactgattaagaagtaggccaactgctggttcgtatgtacaacgtggaagccaagcgactgaacgatgaggctcgtaaagaggccacacagtcacgcgctctggcgattcgctccaacgaactggctgacagtgcatccactaaagttaccgaggctgcccgtgtggcaaaccaagctcaacagctttccaaattctttgagtaatcaaacaggagaaaCCATTATGATCGTTTCTGACATCGAAGCTAACGCCCTCTTAGAGAGCGTCACTAAGt                                                                                                                                                                                                                                                              >  1:815563‑M2/245‑301 (MQ=255)`  
                                               `actaaaggagacacaccatgttcaaactgattaagaagttaggccaactgctggttcgtatgtacaacgtggaagccaagcgactgaacgatgaggctcgtaaagaggccacacagtcacgcgctctggcgattcgctccaacgaactggctgacagtgcatccactaaagttaccgaggatgcccgtgtggcaaaccaagctcaacagctttccaaattctttgagtaatcaaacaggagaaaTCAATATGATCGTTTCTGACATCGAAGctaacgccctcttagagctgtctcttatac                                                                                                                                                                                                                                                              >  1:429102‑M2/245‑288 (MQ=255)`  
                                                `ctaaaggagacacaccttgttcaaactgattaagaagttaggccaactgctggttcgtatgtacaacgtggaagccaagcgactgaacgatgaggctcgtaaagaggccacacagtcacgcgctctggcgattcgctccaacgaactggctgacagtgcatccactaaagttaccgaggctgcccgtgtggcaaaccaagctcaacagctttccaaattctttgagtaatcaaacaggagaaaTCAATATGATCGTTTCTGACATCGAAgctaacgccctcttagagctgtctcttataca                                                                                                                                                                                                                                                             >  1:317954‑M2/244‑287 (MQ=255)`  
                                                `ctaaaggagacacaccatgttcaaactgattaagaagttaggccaactgctggttcgtatgtacaacgtggaagccaagcgactgaacgatgaggctcgtaaagaggccacacagtcacgcgctctggcgattcgctccaacgaactggctgacagtgcatccactaaagttacagaggctgcccgtgtggcaaaccaagcttaacagctttccaaattctttgagtaatcaaacaggagaaaTCAATATGATCGTTTCTGCCATCGAAGCTAACGACCTC‑‑AGAGAGCGtacctaatctcc                                                                                                                                                                                                                                                           >  1:79160‑M2/244‑296 (MQ=255)`  
                                                  `aaaggagacacaccatgttcaaactgattaagaagttaggccaactgctggttcgtatgtacaacgtggaagccaagcgactgaacgatgaggctcgtaaagaggccacacagtcacgcgctctggcgattcgctccaacgaactggctgacagtgcatccactcaagttaccgaggctgcgcgtgtggcaaaccaagctcaacagctttccaaattctttgagtaatcaaacaggagaaaTCAATATGATCGTTTCTGACATCGaagctaacgccctcttagagctgtctctgagacaca                                                                                                                                                                                                                                                           >  1:762981‑M2/242‑285 (MQ=255)`  
                                                       `gagacacaccatgttcaaactgattaagaagttaggccaactgctggttcgtatgtacaacgtggaagccaagctactgaacgatgaggctcgtaaagaggccacacagtcacgcgctctggcgattcgctccaacgaactggctgacagtgcatccactaaagttaccgaggctgaccgtgtggcaaaccaagctcaacagctttccaaattctttgagtaatcaataggagaaaTCAATATGATCGTTTCTGACATCGAAGCTAACGCCCTCTTAGAGAGCGTCACTAAGTTCCACTGc                                                                                                                                                                                                                                                      >  1:652268‑M2/237‑301 (MQ=255)`  
                                                        `gacacaccatgttcaaactgattaagaagttaggccaactgctggttcgtatgtacaacgtggaagccaagcgactgaacgatgaggctcgtaaagaggccacacagtcacgcgctctggcgattcgctccaacgaactggctgacagtgcatccactaaagttaccgaggctgcccgtgtggcaaaccaagctcaacagctttccaaattctttgagtaatcaaacaggagaaaCCATTATGATCGTTTCTGACATCGAAGCTAACTCCCTCTTAGAGAGCGTCACTAAGTTCCACTGCg                                                                                                                                                                                                                                                     >  1:684895‑M2/236‑301 (MQ=255)`  
                                                         `acacaccatgttcaaactgattaagaagttaggccaactgcgggttcgtatgtacaacgtggaagccaagcgactgaacgatgaggctcgtaaagaggccacacagtcacgcgctctggcgattcgctcacacgaactggctgacagtgcatccactaaagttaccgaggctgcccgtgtggcaaaccaaggtcaacagatttccaaattctttgagtaatcaaacatgagaaaTCAATATGATCGTCTCTGACATCGTAGCTAACTCCCCTGTCGATAGCATCACTAAGTTcaacttgtg                                                                                                                                                                                                                                                    >  1:402734‑M2/235‑297 (MQ=255)`  
                                                                `atgttcaaactgattaagaagttaggccaactgctggttcgtatgtacaacgtggaagccaagcgactgaacgatgaggctcgtaaagaggccacacagtcacgcgctctggcgattcgctccaacgaactggctgacagtgcatccactaaagttaccgaggctgcccgtgtggcaaaccaagctcaacagctttccaaattctttgagtaatcaaacaggagaaaTCAATATGATCGTTTCTGACATCGAAGCTAACGCCCTCTTAGAGAGCGTCACTAAGTTCCACTGCGGGGTTATc                                                                                                                                                                                                                                             >  1:216308‑M2/228‑301 (MQ=255)`  
                                                                 `tgttcaaactgattaagaagttaggccaactgctggttcgtatgtacaacgtggaagccaagcgactgaacgatgaggctcgtaaagaggccacacagtcacgcgctctggctattcgctccaacgaactggctgacagtgcatccactaaagttaccgagactgcccgtgttgccaaccaagctcaacagctttccaaattctttgagtaatcaaacaggagaaaCCATTATGATAGTTTCTGACATAGAATCTAACGCCCTCTTAGAGATCGTCACTAAGTTCCACTGCGTGGTTATCt                                                                                                                                                                                                                                            >  1:223316‑M2/227‑301 (MQ=255)`  
                                                                   `ttcaaactgattaagaagttaggccaactgctggttcgtatgtacaacgtggaagccaagcgactgaacgatgaggctcgtaaagaggccacacagtcacgcgctctggcgattcgctccaacgaactggctgacagtgcatccactaaagttaccgaggctgcccgtgtggcaaaccaagctcaacagctttccaaattctttgagtaatcaaacaggagaaaTCAATATGATCGTTTCTGACATCGAAGCTAACGCCCTCTTAGAGAGCGTCACTAAGTTCCACTGCGGGGTTATCTAc                                                                                                                                                                                                                                          <  1:691212‑M2/77‑1 (MQ=255)`  
                                                                    `tcaaattgattaagaagttaggccaactgctggttcgtatgtacaacgtggaagccaagcgactgaacgatgagtctcgtaaagaggccacacagtcacgcgctctggcgattcgctccaacgaactggctgacagtgcatccactaaagttaccgaggctgcccgtgtggcaaaccaagctcaacagctttccaaattctttgagtaatcaaacaggagaaaTCAATATGATCGTTTCTGAAATCGAAGCTAACGCCCTCTTAGAGAGCGTCACTAAGTTCCACTGCGGGGTTATCTACg                                                                                                                                                                                                                                         <  1:195067‑M2/78‑1 (MQ=255)`  
                                                                            `attaagaagttaggccaactgctggttcgtatgtacaacgtggaagccaagcgactgaacgatgaggctcgtaaagaggccacacagtcacgcgctctggcgattcgctccaacgaactggctgacagtgcctccactaaagttaccgaggctgcccgtgtggcaaaccaagcccaacagcgttccaaattctttgagtaatcaaacaggagaaaTCAATATGATCGTTTCTGACATCGAAGCTAACGCCCTCTTAGAGAGCGTCACTAAGTTCCACTGCGGGGTGATCTACGACTACTcc                                                                                                                                                                                                                                 >  1:569559‑M2/216‑301 (MQ=255)`  
                                                                             `attcagaagttaggccaactgctggttcgtatgtacaacgtggaagccaagcgactgaacgatgaggctcgtaaagaggccacacagtcacgcgctctggcgattcgctccaacgaactggctgacagtgcatccactaaagttaccgaggctgcccgtgtggcaaaccaagctcaacagctttccaaattctttgagtaatcaataggagaaaTCAATATGATCGTTTCTGACATCGAAGCTAACGCCCTCTTAGAGAGCGTCACTAAGTTCCACTGCGGGGTTATCTACGACTACTCCa                                                                                                                                                                                                                                >  1:11424‑M2/215‑301 (MQ=255)`  
                                                                                        `ataagagacagggttcgtatgtacaacgtggaagccaagcgactgaacgatgaggctcgtaaagaggccacacagtcacgcgctctggcgattcgctccaacgaactggctgacagtgcatccactaaagttaccgaggctgcccgtgtggcaaaccaagctcaacagctttccaaattctttgagtaatcaaacaggagaaaTCAATATGATCGTTTCTGACATCGAAGCTAACGCCCTCTTAGAGAGCGTCACTAAGTTCCACTGCGGGGTTATCTACGACTACTCCACCGCTGAGTAc                                                                                                                                                                                                                     <  1:51272‑M2/98‑1 (MQ=255)`  
                                                                                           `ttataagagacaggtatgttcaccgtggaatccacgcgccttaacgattatgctcttaaagaggccacacagtcacgcgctctggcgattcgctccatcgaactggctgaccgtgcatccactaaagttaccgaggctgcccttgtggcaaaccaagctcaacagctttccaaattctttgggtaatcatacaggagaaaTCAATTTGATCGTTTCTTACATCGAAGCTAACGCACTCTTAGAGAGCGTCACTAAGTTCCACTGCGGGGTTATCTACGACTACTCCACCGCTGAGTACGTa                                                                                                                                                                                                                  <  1:266399‑M2/101‑1 (MQ=255)`  
                                                                                             `actgctggttcgtatgtacaacgtggaagccaagcgactgaacgatgaggctcgtaaagaggccacacagtcacgcgctctggcgattcgctccaacgaactggctgacagtgcatccactaaagttaccgaggctgcccgtgtggcaaaccaagctcaacagctttccaaattctttgagtaatcaaacaggagaaaTCAATATGATCGTTTCTGACATCGAAGCTAACGCCCTCTTAGAGAGCGTCACTAAGTTCCACTGCGGGGTTATCTACGACTACTCCACCGCTGAGTACGTAAg                                                                                                                                                                                                                <  1:712615‑M2/103‑1 (MQ=255)`  
                                                                                              `ctgctggttcgtatgtacaacgtggaagccaagcgactgaacgatgaggctcgtaaagaggccacacagtcacgcgctctggcgattcgctccaacgaactggctgacagtgcatccactaaagttaccgaggctgcccgtgtggcaaaccaagctcaacagctttccaaattctttgagtaatcaaacaggagaaaTCAATATGATCGGTTCTGACATCGAAGCTAACGCCCTCTTAGAGAGCGTCACTAAGTTCAACTGCGGGGTTATCTACGACTACTCCACCGCTGAGTACGTAaga                                                                                                                                                                                                               >  1:370058‑M2/198‑300 (MQ=255)`  
                                                                                              `ctgctggttcgtatgtacaacgtggaagccaagcgactgaacgatgaggctcgtaaaaatgccacacagtcacgcgctctggcgattcgctccaacgaactggctgccagtgcatccactaccgttcccgaggctgcccgtgtggcaaaccaagctcaacagctttccaaattctttgagtaatcaaacaggagacaTCAATATGATCGTTTCTGACATCGAAGCTAACGCACTCTTATAGAGCGTCACTAAGTTCCACTTCGGGGTTATCTACGACTACTCCACCGCTGAGTACGTCAGc                                                                                                                                                                                                               >  1:475947‑M2/198‑301 (MQ=255)`  
                                                                                                 `gatgtgtataagagacaggtggaagccaagcgactgaacgatgaggctcgtaaagaggccacacagtcacgcgctctggcgattcgctccaacgaactggctgacagtgcatccactaaagttaccgaggctgcccgtgtggcaaaccaagctcaacagctttccaaattctttgagtaatcaaacaggagaaaTCAATATGATCGTTTCTGACATCGAAGCTAACGCCCTCTTAGAGAGCGTCACTAAGTTCCACTGCGGGGTTATCTACGACTACTCAACCGCTGAGTACGTAAGCTAc                                                                                                                                                                                                            <  1:770094‑M2/107‑1 (MQ=255)`  
                                                                                                       `cgtatgtacaacgtggaagccaagcgactgaacgatgaggctcgtaaagaggccacacagtcacgcgctctggcgattcgctccaacgaactggctgacagtgcatccactaaagttaccgaggctgcccgtgtggcaaaccaagctcaacagctttccaaattctttgagtaatcaaacaggagaaaTCAATATGATCGTTTCTGACATCGAAGCTAACGCCCTCTTAGAGAGCGTCACTAAGTTCCACTGCGGGGTTATCTACGACTACTCCACCGCTGAGTACGTAAGCTACCGTCCg                                                                                                                                                                                                      >  1:143082‑M2/189‑301 (MQ=255)`  
                                                                                                       `cgtatgtacaacgtggaagccaagcgactgaacgatgaggctcgtaaagaggccacacagtcacgcgctctggcgattcgctccaacgaactggctgacagtgcatccactaaagttaccgaggctgcccgtgtggcaaaccaagctcaacagctttccaaattctttgagtaatcaaacaggagaaaTCAATATGATCGTTTCTGACATCGAAGCTAACGCCATCTTAGAGAGCGTCACTAAGTTCCACTGCGGGGTTATCTACGACTACTCCACCGCTGAGTACGTAAGCTACCGTCCg                                                                                                                                                                                                      >  1:386377‑M2/189‑301 (MQ=255)`  
                                                                                                       `cgtatgtacaacgtggaagcaaagcgactgaacgatgaggctcgtaaagaggccacacagtcacgcgctctggcgattcgctccaacgaactggctgacagtgcatccactaaagttaccgaggctgcccgtgtggcaaaccaagctcaacagctttccaaattctttgagtaatcaaacaggagaaaTCAATATGATCGTTTCTGACATCGAAGCTAACGCCCTCTTAGAGAGCGTCACTAAGTTCCACTGCGGGGTTATCTACGACTACTCCACCGCTGAGTACGTAAGCTACCGTCCg                                                                                                                                                                                                      >  1:784638‑M2/189‑301 (MQ=255)`  
                                                                                                           `tgtacaacgtggaagccaagcgactgaacgatgaggctcgtaaagaggccacacagtcacgcgctctggcgattcgctccaacgaactggctgacagtgcatccactaaagttaccgaggctgcccgtgtggcaaaccaagctcaacagctttccaaattctttgagtaataaaacaggagaaaTCAATATGATCGTTTCTGACATCGAAGCTAACGCCCTCTTAGAGAGCGTCACTAAGTTCCACTGCGGGGTTATCTACGACTACTCCACCGCTGAGTACGTAAGCTACCGTCCGAGTg                                                                                                                                                                                                  <  1:436024‑M2/117‑1 (MQ=255)`  
                                                                                                                     `gtaagccaaaggactgaaagatgaggcccgtaaatatgccaaacagtcacacgctctggctattcgctccaacgaactggctgacagtgcatccactaaagttaccgaggcttcccgtgtggcaaaccaagctcaacagctttccaaattctttgagtaatctaacaggataaaTCAATATGATCGTTTCTGACATCGAAGCTAACGCCCTCTTAGAGAGCGTCACTAAGTTCCACTGCGGGGTTATCTACGACTACTCCACCGCTGAGTACGTAAGCTACCGTCCGAGTGACTTCGGTGc                                                                                                                                                                                        <  1:108489‑M2/127‑1 (MQ=255)`  
                                                                                                                      `gaagccaagcgactgaacgatgaggctcgtaaagaggccacacagtcacgcgctctggcgattcgctccaacgaactggctgacagtgcatccactaaagttaccgaggctgcccgtgtggcaaaccaagctcaacagctttccaaattctttgagtaatcaaacaggagaaaTCAATATGATCGTTTCTGACATCGAAGCTAACGCCCTCTTAGAGAGCGTCACTAAGTTCCACTGCGGGGTTATCTACGACTACTCCACCGCTGAGTACGTAAGCTACCGTCCGAGTGACTTCGGTGCg                                                                                                                                                                                       <  1:629730‑M2/128‑1 (MQ=255)`  
                                                                                                                              `gcgactgaacgatgaggctcgtaaagaggccacacagtcacgcgctctggcgattcgctccaacgaactggctgacagtgcatccactaaagttaccgaggctgcccgtgtggcaaaccaagctcaacagctttccaaattctttgagtaatcaaacaggagaaaTCAATATGATCGTTTCTGACATCGAAGCTAACGCCCTCTTAGAGAGCGTCACTAAGTTCCACTGCGGGGTTATCTACGACTACTCCACCGCTGAGTACGTAAGCTACCGTCCGAGTGACTTCGGTGCGTATCTGGa                                                                                                                                                                               <  1:21029‑M2/136‑1 (MQ=255)`  
                                                                                                                                    `gaacgatgaggctcgtaaagaggccacacagtcacgcgctctggcgattcgctccaacgaactggctgacagtgcatccactaaagttaccgaggctgcccgtgtggcaaaccaagctcaacagctttccaaattctttgagtaatcaaacaggagaaaCCAATATGATCGTTTCTGACATCGAAGCTAACGCCCTCTTAGAGAGCGTAACTAAGTTCCACTGCGGGGTTATCTACGACTACTCCACCGCTGAGTACGTAAGCTACCGTCCGAGTGACTTcggtgcgtatcctgtctcttt                                                                                                                                                                         >  1:593190‑M2/160‑291 (MQ=255)`  
                                                                                                                                               `ctcgtaaagaggccacacagtcacgcgctctggcgattcgctccaacgaactggctgacagtgcatccactaaagttaccgaggctgcacgtgtggcaaaccaagctcaacagctttccaaattctttgagtaatcaaacaggagaaaTCAATATGATCGTTTCTGACATCGAAGCTAACGCCCTCTTAGAGAGCGTCACTAAGTTCCACTGCGGGGTTATCTACGACTACTCCACCGCTGAGTACGTAAGCTACCGTCCGAGTGACTTCGGTGCGTATCTGGATGCGCTGGAAGCCGAgg                                                                                                                                                              >  1:452799‑M2/149‑301 (MQ=255)`  
                                                                                                                                                       `gaggccacacagtcacgcgctctggcgattcgctccaacgaactggctgacagtgcatccactaaagttaccgaggctgcccgtgtggcaaaccaagctcaacagctttccaaattctttgagtaatcaaacaggagaaaTCAATATGATCGTTTCTGACATCGAAGCTAACGCCCTCTTAGAGAGCGTCACTAAGTTCCACTGCGGGGTTATCTACGACTACTCCACCGCTGAGTACGTAAGCTACCGTCCGAGTGACTTCGGTGCGTATCTGGATGCGCTGGAAGCCGAGGTTGCAcgg                                                                                                                                                      >  1:362183‑M2/141‑300 (MQ=255)`  
                                                                                                                                                         `ggccacacagtcacgcgctctggcgattcgctccaacgaactggctgacagtgcatccactaaagttaccgaggctgcccgtgtggcaaaccaagctcaacagctttccaaattctttgagtaatcaaacaggagaaaTCAATATGATCGTTTCTGACATCGAAGCTAACGCCCTCTTAGAGAGCGTCACTAAGTTCCACTGCGGGGTTATCTACGACTACTCCACCGCTGAGTACGTAAGCTACCGTCCGAGTGACTTCGGTGCGTACCTGGATGCGCTGGAAGCCGAGGTTGCACGAgg                                                                                                                                                    >  1:736734‑M2/139‑301 (MQ=255)`  
                                                                                                                                                                            `ctggcgattcgctccaacgaactggctgacagtgcatccactaaagttaccgaggctgcccgtgtggcaaaccaagctcaacagctttccaaattctttgagtaatcaaacaggagaaaTCAATATGATCGTTTCTGACATCGAAGCTAACGCCCTCTTAGAGAGCGTCACTAAGTTCCACTGCGGGGTTATCTACGACTACTCCACCGCTGAGTACGTAAGCTACCGTCCGAGTGACTTCGGTGCGTATCTGGATGCGCTGGAAGCCGAGGTTGCACGAGGCGGTCTTATTGTGTTCcac                                                                                                                                 <  1:311691‑M2/182‑1 (MQ=255)`  
                                                                                                                                                                              `ggtgattcgctccaacgaactggctgacagtgcatccactaaagttaccgaggctgcccgtgtggcaaaccaagctcaacagctttccaaattctttgagtaatcaaacaggagaaaTCAATATGATCGTTTCTGACATCGAAGCTAACGCCCTCTTAGAGAGCGTCACTAAGTTCCACTGCGGGGTTATCTACGACTACTCCACCTCTGAGTACGTAAGCTACCGTCCGAGTGACTTCGGTGCGTATCTGGATGCGCTGGAAGCCGAGGTTGCACGAGGCGGTCTTATTGTGTTCCACaa                                                                                                                               >  1:498028‑M2/118‑301 (MQ=255)`  
                                                                                                                                                                                  `attcgctccaacgaactggctgacagtgcatccactaaagttaccgaggctgcccgtgtggcaaaccaagctcaacagctttccaaattctttgagtaatcaaacaggagaaaTCAATATGATCGTTTCTGACATCGAAGCTAACGCCCTCTTAGAGAGCGTCACTAAGTTCCACTGCGGGGTTATCTACGACTACTCCACCGCTGAGTACGTAAGCTACCGTCCGAGTGACTTCGGTGCGTATCTGGATGCGCTGGAAGCCGAGGTTGCACGAGGCGGTCTTATTGTGTTCCACAACGGt                                                                                                                           <  1:158126‑M2/188‑1 (MQ=255)`  
                                                                                                                                                                                      `gctccaacgaactggctgacagtgcatccactaaagttaccgaggctgcccgtgtggcaaaccaagctcaacagctttccaaattctttgagtaatcaaacaggagaaaTCAATATGATCGTTTCTGACATCGAAGCTAACGCCCTCTTAGAGAGCGTCACTAAGTTCCACTGCGGGGTTATCTACGACTACTCCACCGCTGAGTACGTAAGCTACCGTCCGAGTGACTTCGATGCGTATCTGGATGCGCTGGAAGCCGAGGTtacacgaggcggtcttattgctgtctctcatacacatt                                                                                                                       >  1:173976‑M2/110‑283 (MQ=255)`  
                                                                                                                                                                                        `tccaacgaactggctgacagtgcatccactaaagttaccgaggctgcccgtgtggcaaaccaagctcaacagctttccaaattctttgagtaatcaaacaggagaaaTCAATATGATCGTTTCTGACATCGAAGCTAACGCCCTCTTAGAGAGCGTCACTAAGTTCCACTGCGGGGTTATCTACGACTACTCCACCGCTGAGTACGTAAGCTACCGTCCGAGTGACTTCGGTGCGTATCTGGATGCGCTGGAAGCCGAGGTTGCACGAGGCGGTCTTATTGTGTTCCACAACGGTCACAAg                                                                                                                     <  1:37918‑M2/194‑1 (MQ=255)`  
                                                                                                                                                                                        `cccaacgaactggctgacagtgcatccactaaagttaccgaggctgcccgtgtggcaaaccaagctcaacagctttccaaattctttgagtaatcaaacagtagaaaTCAACATGATCGTTTCTGACATCGAAGCTAACGCCCTCTTAGAGAGCGTCACTAAGTTCCACTGCGGGGTTATCTACGACTACTCCACCGCCGAGTACGTAAGCTACCGTCCGAGTGACTTCGGTGCGTATCTGGATGCGCTGGAAGCCGAGGTTGCACGAGGCGGTCTTATTGTGTTCCACAACGGTCACAAg                                                                                                                     <  1:13598‑M2/194‑1 (MQ=255)`  
                                                                                                                                                                                           `agagacagggctgacagtgcatccactaaagttaccgaggatgcccgtgtggcaaaccaagctcaatagctttccaaattctttgagtaatcaaacaggagaaaTCAATATGATCGTTTCTGACATCGAAGCTAACGCCCTCTTAGAGAGCGTCACTAAGTTCCACTGCGGGGTTATCTACGACTACTCCACCGCTGAGTACGTAAGCTACCGTCCGAGTGACTTCGGTGCGTATCTGGATGCGCTGGAAGCCGAGGTTGCACGAGGCGGTCTTATTGTGTTCCACAACGGTCACAAGTAt                                                                                                                  <  1:518621‑M2/197‑1 (MQ=255)`  
                                                                                                                                                                                             `aaagagacaggacagtgcatccactaaagttaccgaggctgcccgtgtggcaaaccaagctcaacagctttccaaattctttgagtaatcaaacaggagaaaTCAATATGATCGTTTCTGACATCGAAGCTAACGCCCTCTTAGAGAGCGTCACTAAGTTCCACTGCGGGGTTATCTACGACTACTCCACCGCTGAGTACGTAAGCTACCGTCCGAGTGACTTCGGTGCGTATCTGGATGCGCTGGAAGCCGAGGTTGCACGAGGCGGTCTTATTGTGTTCCACAACGGTCACAAGTATGa                                                                                                                <  1:601283‑M2/199‑1 (MQ=255)`  
                                                                                                                                                                                               `gaactggctgacagtgcatccactaaagttaccgaggctgcccgtgtggcaaaccaagctcaacagctttccaaattctttgagtaatcaataggagaaaTCAATATGATCGTTTCTGACATCGAAGCTAACGCCCTCTTAGAGAGCGTCACTAAGTTCCACTGCGGGGTTATCTACGACTACTCCACCGCTGATTACGTAAGCTACCGTCCGAGTGACTTCGGTGCGTCTCTGGATGCGCTGGAAGCCGAGGTTGCCAGAGGCGGTCTTATTGTGTTCCACACCGGTCACAAGTATGACg                                                                                                              >  1:777996‑M2/101‑301 (MQ=255)`  
                                                                                                                                                                                                `actggctgacagtgcatccactaaagttaccggggctggccgtgtggcaaaacaagctcaacagcgttccaaattctttgagtaatcaaacaggagaaaTCAATATGATGGTTTCTGACATCGAAGCTAACGCCCTCTTAGAGGTCGTCACTAAGTTCCACTGCGGGGTTATCTACGACTGCGCCACCGCTAAGTACGTAAGCTACCGTCCGAGTGACTTCGGTGCGTATCTGGATGCGCTGGAAGCCGAGGTTGCACGAGGCGGTCTTATTGTGTTCCACAACGGTCACAAGTATGACGt                                                                                                             <  1:822230‑M2/202‑1 (MQ=255)`  
                                                                                                                                                                                                 `ctggctgacagtgcatccactaaagttaccgaggctgcccgtgtggcaaaccaagctcaacagctttccaaattctttgagtaatcaaacaggagaaaTCAATATGATCGTTTCTGACATCGAAGCTAACGCCCTCTTAGAGAGCGTCACTAAGTTCCACTGCGGGGTTATCTACGACTACTCCACCGCTGAGTACGTAAGCTACCGTCCGAGTGACTTCGGTGCGTATCTGGATGCGCTGGAAGCCGAGGTTGCACGAGGCGGTCTTATTGTGTTCCACAACGGTCACAAGTATGACGtt                                                                                                            >  1:303613‑M2/99‑301 (MQ=255)`  
                                                                                                                                                                                                 `ctggctgacagtgcatccactaaagttaccgaggctgcccgtgtggcaaaccaagctcaacagctttccaaattctttgagtaatcaaacaggagaaaTCAATATGATCGTTTCTGACATCGAAGCTAACGCCCTCTTAGAGAGCGTCACTAAGTTCCACTGCGGGGTTATCTACGACTACTCCACCGCTGAGTACGTAAGCTACCGTCCGAGTGACTTCGGTGCGTATCTGGATGCGCTGGAAGCCGAGGTTGCACGAGGCGGTCTTATTGTGTTCCACAACGGTCACAAGTATGACGtt                                                                                                            >  1:353153‑M2/99‑301 (MQ=255)`  
                                                                                                                                                                                                   `gtgggctcggagatgtgtataagagacagcacgaaagttaccgaggctgcccgtgtggcaaaccaagctcaacagctttccaaattctttgagtaaTCAATATGATCGTTTCTGACATCGAAGCTAACGCCCTCTTAGAGAGCGTCACTAAGTTCCACTGCGGGGTTATCTACGACTACTCCACCGCTGAGTACGTAAGCTACCGTCCGAGTGACTTCGGTGCGTATCTGGATGCGCTGGAAGCCGAGGTTGCACGAGGCGGTCTTATTGTGTTCCACAACGGTCACAAGTATGACGTTcc                                                                                                          <  1:728189‑M2/205‑1 (MQ=255)`  
                                                                                                                                                                                                    `ggctgacagtgcatccactaaagttaccgaggctgcccgtgtggcaaaccaagctcaacagctttccaaattctttgagtaatcaataggagaaaTCAATATGATCGTTTCTGACATCGAAGCTAACGCCCTCTTAGGGAGCGTCACTAAGTTCCACTGCGGGGTTATCTACGACTACTCCACCGCTGAGTACGTAAGCTACCGTCCGAGTGACTTCGGTGCGTATCTGGATGCGCTGGAAGCCGAGGTTGCACGAGGCGGTCTTATTGTGTTCCACAACGGTCACAAGTATGACGTTCCt                                                                                                         >  1:345094‑M2/96‑301 (MQ=255)`  
                                                                                                                                                                                                      `ggacagtgcatccactaaagttaccgaggctgcccgtgtggcaaaccaagctcaacagctttccaaattctttgagtaatcaaacaggagaaaTCAATATGATCGTTTCTGACATCGAAGCTAACGCCCTCTTAGAGAGCGTCACTAAGTTCCACTGCGGGGTTATCTACGACTACTCCACCGCTGAGTACGTAAGCTACCGTCCGAGTGACTTCGGTGCGTATCTGGATGCGCTGGAAGCCGAGGTTGCACGAGGCGGTCTTATTGTGTTCCACAACGGTCACAAGTATGACGTTCCTGc                                                                                                       <  1:577804‑M2/208‑1 (MQ=255)`  
                                                                                                                                                                                                         `cagtgcatccactaaagttaccgaggctgcccgtgtggcaaaccaagctcaacagctttccaaattctttgagtaatcaaacaggagaaaTCAATATGATCGTTTCTGACATCGAAGCTAACGCCCTCTTAGAGAGCGTCACTAAGTTCCACTGCGGGGTTATCTACGACTACTCCACCGCTGAGTACGTAAGCTACCGTCCGAGTGACTTCGGTGCGTATCTGGATGCGCTGGAAGCCGAGGTTGCACGAGGCGGTCTTATTGTGTTCCACAACGGTCACAAGTATGACGTTCCTGCAtt                                                                                                    >  1:229489‑M2/91‑301 (MQ=255)`  
                                                                                                                                                                                                           `gtgcatccactaaagttaccgaggctgcccgtgtggcaaaccaagctcaacagctttccaaattctttgagtaatcaaacaggagaaaTCAATATGATCGTTTCTGACATCGAAGCTAACGCCCTCTTAGAGAGCGTCACTAAGTTCCACTGCGGGGTTATCTACGACTACTCCACCGCTGAGTACGTAAGCTACCGTCCGAGTGACTTCGGTGCGTATCTGGATGCGCTGGAAGCCGAGGTTGCACGAGGCGGACTTATTGTGTTCCACAACGgtcacaagtatgactgtctcttatacc                                                                                                  >  1:483113‑M2/89‑288 (MQ=255)`  
                                                                                                                                                                                                                       `aagttaccgaggctgcccgtgtggcaaaccaagctcaacagctttccaaattctttgagtaatcaaacaggagaaaTCAATATGATCGTTTCTGACATCGAAGCTAACGCCCTCTTAGAGAGCGTCACTAAGTTCCACTGCGGGGTTATCTACGACTACTCCACCGCTGAGTACGTAAGCTACCGTCCGAGTGACTTCGGTGCGTATCTGGATGCGCTGGAAGCCGAGGTTGCACGAGGCGGTCTTATTGTGTTCCACAACGGTCACAAGTATGACGTTCCTGCATTGACCACACTGGCaa                                                                                      >  1:52675‑M2/77‑301 (MQ=255)`  
                                                                                                                                                                                                                        `agttaccgaggctgcccgtgtggcaaaccaagctcaacagctttccaaattctttgagtaatcaaacaggagaaaTCAATATGATCGTTTCTGACATCGAAGCTAACGCCCTCTTAGAGAGCGTCACTAAGTTCCACTGCGGGGTTATCTACGACTACTCCACCGATGAGTACGTAAGCTACCGTCCGAGTGACTTCGGTGCGTATCTGGATGCGCTGGAAGCCGAGGTTGCACGAGGCGGTCTTATTGTGTTCCACAACGGTCACAAGTATGACGTTCCTGCATTGACCAAACTGGCaaa                                                                                     >  1:182057‑M2/76‑301 (MQ=255)`  
                                                                                                                                                                                                                               `gaggctgcccgtgtggcaaaccaagctcaacagctttccaaattctttgagtaatcaaacaggagaaaCCATTATGATCGTTTCTGACATCGAAGCTAACGCCCTCTTAGAGAGCGTCACTAAGTTCCACTGCGGGGTTATCTACGACTACTCCACCGCTGAGTACGTAAGCTACCGTCCGAGTGACTTCGGTGCGTATCTGGATGCGCTGGAAGCCGAGGTTGCACGAGGCGGTCTTATTGTGTTCCACAACGGTCACAAGTATGACGTTCCTGCATTGACCAAACTGGCAAAGTTGCaa                                                                              >  1:189462‑M2/69‑301 (MQ=255)`  
                                                                                                                                                                                                                                           `gtggcaaaccaagctcaacagctttccaaattctttgagtaatcaaacaggagaaaTCAATATGATCGTTTCTGACATCGAAGCTAACGCCCTCTTAGAGAGCGTCACTAAGTTCCACTGCGGGGTTATCTACGACTACTCCACCGCTGAGTACGTAAGCTACCGTCCGAGTGACTTCGGTGCGTATCTGGATGCGCTGGAAGCCGAGGTTGCACGAGGCGGTCTTATTGTGTTCCACAACGGTCACAAGTATGACGTTCCTGCATTGACCAAACTGGCAAAGTTGCAATTGAAccgaggg                                                                  >  1:490232‑M2/57‑299 (MQ=255)`  
                                                                                                                                                                                                                                                  `accaagctcaacagctttccaaattctttgagtaatcaaacaggagaaaTCAATATGATCGTTTCTGACATCGAAGCTAACGCCCTCTTAGAGAGCGTCACTAAGTTCCACTGCGGGGTTATCTACGACTACTCCACCGCTGAGTACGTAAGCTACCGTCCGAGTGACTTCGGTGCGTATCTGGATGCGCTGGAAGCCGAGGTTGCACGAGGCGGTCTTATTGTGTTCCACAACGGTCACAAGTATGACGTTCCTGCATTGACCAAACTGGCAAAGTTGCAATTGAACCGAGAGTTCCAcc                                                           >  1:281046‑M2/50‑301 (MQ=255)`  
                                                                                                                                                                                                                                                   `ccaagctcaacagctttccaaattctttgagtaatcaaacaggagaaaTCAATATGATCGTTTCTGACATCGAAGCTAACGCCCTCTTAGAGAGCGTCACTAAGTTCCACTGCGGGGTTATCTACGACTACTCCACCGCTGAGTACGTAAGCTACCGTCCGAGTGACTTCGGTGCGTATCTGGATGCGCTGGAAGCCGAGGTTGCACGAGGCGGTCTTATTGTGTTCCACAACGGTCACAAGTATGACGTTCCTGCATTGACCAAACTGGCAAAGTTGCAATTGAACCGAGAGTTCCACCt                                                          <  1:808244‑M2/253‑1 (MQ=255)`  
                                                                                                                                                                                                                                                     `aagctcaacagctttccaaatgcattgagtaatcaaacaggagaaaTCAATATGATCGTTTCTGACATCGAAGCTAACGCCCTCTTAGAGAGCGTCACTAAGTTCCACTGCGGGGTTATCTACGACTACTCCACCGCTGAGTACGTAAGCTACCGTCCGAGTGACTTCGGTGCGTATCTGGATGCGCTGGAAGCCTAGGTTGCACGAGGCGGTCTTATTGTGTTCCACAACGGTCACAAGTATGACGTTCCTGCATTGACCAAACTGGCAAAGTTGCAATTGAACCGAGAGTTCCACCTTc                                                        <  1:295744‑M2/255‑1 (MQ=255)`  
                                                                                                                                                                                                                                                       `gctcaacagctttccaaattctttgagtaatcaaacaggagaaaTCAATATGATCGTTTCTGACATCGAAGCTAACGCCCTCTTAGAGAGCGTCACTAAGTTCCACTGCTGGGTTATCTACGACTACTCCACCGCTGAGTACGTAAGCTACCGTCCGAGTGACTTCGGTGCGTATCTGGATGCGCTGGAAGCCGAGGTTGCACGAGGCGGTCTTATTGTGTTCCACAACGGTCACAAGTATGACGTTCCTGCATTGACCAAACTGGCAAAGTTGCAATTGAACCGAGAGTTCcacctctgt                                                      >  1:127776‑M2/45‑297 (MQ=255)`  
                                                                                                                                                                                                                                                                `aaagagacagtctttgagtaatcaaacaggagaaaTCAATATGATCGTTTCTGACATCGAAGCTAACGCCCTCTTAGAGAGCGTCACTAAGTTCCACTGCGGGGTTATCTACGACTACTCCACCGCTGAGTACGTAAGCTACCGTCCGAGTGACTTCGGTGCGTATCTGGATGCGCTGGAAGCCGAGGTTGCACGAGGCGGTCTTATTGTGTTCCACAACGGTCACAAGTATGACGTTCCTGCATTGACCAAACTGGCAAAGTTGCAATTGAACCGAGAGTTCCACCTTCCTCGTGAGAAc                                             <  1:421028‑M2/266‑1 (MQ=255)`  
                                                                                                                                                                                                                                                                     `caaattctttgagtaatcaaacaggagaaaTCAATATGATCGTTTCTGACATCGAAGCTAACGCCCTCTTAGAGAGCGTCACTAAGTTCCACTGCGGGGTTATCTACGACTACTCCACCGCTGAGTACGTAAGCTACCGTCCGAGTGACTTCGGTGCGTATCTGGATGCGCTGGAAGCCGAGGTTGCACGAGGCGGTCTTATTGTGTTCCACAACGGTCACAAGTATGACGTTCCTGCATTGACCAAACTGGCAAAGTTGCAATTGAACCGAGAGTTCCACCTTCCTCGTGAGAACTGTAt                                        <  1:704463‑M2/271‑1 (MQ=255)`  
                                                                                                                                                                                                                                                                          `tctttgagtaatcaaacaggagaaaTCAATATGATCGTTTCTGACATCGAAGCTAACGCCCTCTTAGAGAGCGTCACTAAGTTCCACTGCGGGGTTATCTACGACTACTCCACCGCTGAGTACGTAAGCTACCGTCCGAGTGACTTCGGTGCGTATCTGGATGCGCTGGAAGCCGAGGTTGCACGAGGCGGTCTTATTGTGTTCCACAACGGTCACAAGTATGACGTTCCTGCATTGACCAAACTGGCAAAGTTGTAATTGAACCGAGAGTTCCACCTTCCTCGTGAGAACTGTATTGaca                                   <  1:562982‑M2/276‑1 (MQ=255)`  
                                                                                                                                                                                                                                                                            `tttgagtaatcaaacaggagaaaTCAATATGATCGTTTCTGACATCGAAGCTAACGCCCTCTTAGAGAGCGTCACTAAGTTCCACTGCGGGGTTATCTACGACTACTCCACCGCTGAGTACGTAAGCTACCGTCCGAGTGACTTCGGTGCGTATCTGGATGCGCTGGAAGCCGAGGTTGCACGAGGCGGTCTTATTGTGTTCCACAACGGTCACAAGTATGACGTTCCTGCATTGACCAAACTGGCAAAGTTGCAATTGAACCGAGAGTTCCACCTTCCTCGTGAGAACTGTATTGACAcc                                 <  1:201450‑M2/278‑1 (MQ=255)`  
                                                                                                                                                                                                                                                                                  `taatcaaacaggagaaaTCAATATGATCGTTTCTGACATCGAAGCTAACGCCCTCTTAGAGAGCGTCACTAAGTTCCACTGCGGGGTTATCTACGACTACTCCACCGCTGAGTACGTAAGCTACCGTCCGAGTGACTTCGGTGCGTATCTGGATGCGCTGGAAGCCGAGGTTGCACGAGGCGGTCTTATTGTGTTCCACAACGGTCACAAGTATGACGTTCCTGCATTGACCAAACTGGCAAATTTGCAATTGAACCGAGAGTTCCACCTTCCTCGTGAGAACTGTATTGACACCCctgtc                           >  1:332043‑M2/18‑300 (MQ=255)`  
                                                                                                                                                                                                                                                                                  `aaatcaaacaggagaaaTCAATATGATCGTTTCTGACATCGAAGCTAACGCCCTCTTAGAGAGCGTCACTAAGTTCCACTGCGGGGTTATCTACGACTACTCCACCGCTGAGTACGTAAGCTACCGTCCGAGTGACTTCGGTGCGTATCTGGATGCGCTGGAAGCCGAGGTTGCACGAGGCGGTCTTATTGTGTTCCACAACGGTCACAAGTATGACGTTCCTGCATTGACCAAACTGGCAAAGTTGCAATTGAACCGAGAGTTCCACCTTCCTCGTGAGAACTGTATTGACACCCTtgtg                           <  1:676535‑M2/284‑1 (MQ=255)`  
                                                                                                                                                                                                                                                                                   `aatcaaacaggagaaaTCAATATGATCGTTTCTGACATCGAAGCTAACGCCCTCTTAGAGAGCGTCACTAAGTTCCACTGCGGGGTTATCTACGACTACTCCACCGCTGAGTACGTAAGCTACCGTCCGAGTGACTTCGGTGCGTATCTGGATGCGCTGGAAGCCGAGGTTGCACGAGGCGGTCTTATTGTGTTCCACAACGGTCACAAGTATGACGTTCCTGCATTGACCAAACTGGCAAAGTTGCAATTGAACCGAGAGTTCCACCTTCCTCGTGAGAACTGTATTGGCACCCTTgtgt                          <  1:91994‑M2/285‑1 (MQ=255)`  
                                                                                                                                                                                                                                                                                     `taagagacaggaaaTCAATATGATCGTTTCTGACATCGAAGCTAACGCCCTCTTAGAGAGCGTCACTAAGTTCCACTGCGGGGTTATCTACGACTACTCCACCGCTGAGTACGTAAGCTACCGTCCGAGTGACTTCGGTGCGTATCTGGATGCGCTGGAAGCCGAGGTTGCACGAGGCGGTCTTATTGTGTTCCACAACGGTCACAAGTATGACGTTCCTGCATTGACCAAACTGGCAAAGTTGCAATTGAACCGAGAGTTCCACCTTCCTCGTGAGAACTGTATTGACACCCTTGtgttg                        <  1:460681‑M2/287‑1 (MQ=255)`  
                                                                                                                                                                                                                                                                                            `ggagaaaCCATTATGATCGTTTCTGACATCGAAGCTAACGCCCTCTTAGAGAGCGTCACTAAGTTCCACTGCGGGGTTATCTACGACTACTCCACCGCTGAGTACGTAAGCTACCGTCCGAGTGACTTCGGTGCGTATCTGGATGCGCTGGAAGCCGAGGTTGCACGAGGCGGTCTTATTGTGTTCCACAACGGTCACAAGTATGACGTTCCTGCATTGACCAAACTGGCAAAGTTGCAATTGAACCGAGAGTTCCACCTTCCTCGTGAGAACTGTATTGACACCCTTGTGTTGTCACGtt                 >  1:32471‑M2/8‑301 (MQ=255)`  
                                                                                                                                                                                                                                                                                             `gagaaaCCATTATGATCGTTTCTGACATCGAAGCTAACGCCCTCTTAGAGAGCGTCACTAAGTTCCACTGCGGGGTTATCTACGACTACTCCACCGCTGAGTACGTAAGCTACCGTCCGAGTGACTTCGGTGCGTATCTGGATGCGCTGGAAGCCGAGGTTGCACGAGGCGGTCTTATTGTGTTCCACAACGGTCACAAGTATGACGTTCCTGCATTGACCAAACTGGCAAAGTTGCAATTGAACCGAGAGTTCCACCTTCCTCGTGAGAACTGTATTGACACCCTTGTGTTGTCACGttt                >  1:4687‑M2/7‑301 (MQ=255)`  
                                                                                                                                                                                                                                                                                               `gaaaTCAATATGATCGTTTCTGACATCGAAGCTAACGCCCTCTTAGAGAGCGTCACTAAGTTCCACTGCGGGGTTATCTACGACTACTCCACCGCTGAGTACGTAAGCTACCGTCCGAGTGACTTCGGTGCGTATCTGGATGCGCTGGAAGCCGAGGTTGCACGAGGCGGTCTTATTGTGTTCCACAACGGTCACAAGTATGACGTTCCTGCATTGACCAAACTGGCAAAGTTGCAATTGAACCGAGAGTTCCACCTTCCTCGTGAGAACTGTATTGACACCCTTGTGTTGTCACGTTTGa              >  1:273822‑M2/5‑301 (MQ=255)`  
                                                                                                                                                                                                                                                                                               `gaaaTCAATATGATCGTTTCTGACATCGAAGCTAACGCCCTCTTAGAGAGCGTCACTAAGTTCCACTGCGGGGTTATCTACGACTACTCCACCGCTGAGTACGTAAGCTACCGTCCGAGTGACTTCGGTGCGTATCTGGATGCGCTGGAAGCCGAGGTTGCACGAGGCGGTCTTATTGTGTTCCACAACGGTCACAAGTATGACGTTCCTGCATTGACCAAACTGGCAAAGTTGCAATTGAACCGAGAGTTCCACCTTCCTCGTGAGAACTGTATTGACACCCTTGTGTTGTCACGTTTGa              >  1:637483‑M2/5‑301 (MQ=255)`  
                                                                                                                                                                                                                                                                                                 `aaTCAATATGATCGTTTCTGACATCGAAGCTAACGCCCTCTTAGAGAGCGTCACTAAGTTCCACTGCGGGGTTATCTACGACTACTCCACCGCTGAGTACGTAAGCTACCGTCCGAGTGACTTCGGTGCGTATCTGGATGCGCTGGAAGCCGAGGTTGCACGAGGCGGTCTTATTGTGTTCCACAACGGTCACAAGTATGACGTTCCTGCATTGACCAAACTGGCAAAGTTGCAATTGAACCGAGAGTTCCACCTTCCTCGTGAGAATTGTATTGACACCCTTGTGTTGTCACGTTTGAtt            >  1:609292‑M2/3‑301 (MQ=255)`  
                                                                                                                                                                                                                                                                                                 `aaTCAATATGATCGTTTCTGACATCGAAGCTAACGCCCTCTTAGAGAGCGTCACTAAGTTCCACTGCGGGGTTATCTACGACTACTCCACCGCTGAGTACGTAAGCTACCGTCCGAGTGACTTCGGTGCGTATCTGGATGCGCTGGAAGCCGAGGTTGCACGAGGCGGTCTTATTGTGTTCCACAACGGTCACAAGTATGACGTTCCTGCATTGACCAAACTGGCAAAGTTGCAATTGAACCGAGAGTTCCACCTTCCTCGTGAGAACTGTATTGACACCCTTGTGTTGTCACGTTTGAtt            >  1:720518‑M2/3‑301 (MQ=255)`  
                                                                                                                                                                                                                                                                                                 `aaTCAATATGATCGTTTCTGACATCGAAGCTAACGCCCTCTTAGAGAGCGTCACTAAGTTCCACTGCGGGGTTATCTACGACTACTCCACCGCTGAGTACGTAAGCTACCGTCCGAGTGACGTCGGTGCGTATCTGGATGCGCTGGAAGCCGAGGTTGCACGAGGCGGTCTTATTGTGTTACACAACGGTCACAAGTATGACGTTCCTGCATTGACCAAACTGGCAAAGTTGCAATTGAACAGAGAGTTCCACCTTCCTCGTGAGGACTGTATTGACAACCTTGTGTGGTCACGTTTGAtt            >  1:394569‑M2/3‑301 (MQ=255)`  
                                                                                                                                                                                                                                                                                                      `atatGATCGTTTCTGACATCGAAGCTAACGCCCTCTTAGAGAGCGTCACTAAGTTCCACTGCGGGGTTATCTACGACTACTCCACCTCTGAGTACGTAAGCTACCGTCCGAGTGACTTCGGTGCGTATCTGGATGCGCTGGAAGCCGAGGTTGCACGAGGCGGTCTTATTGTGTTCCACAACGGTCACAAGTATGACGTTCCTGCATTGACCAAACTGGCAAAGTTGCAATTGAACCGAGAGTTCCACCTTCCTCGTGAGAACTGTATTGACACCCTTGTGTTGTCACGTTTTattctttt       >  1:540511/1‑300 (MQ=255)`  
                                                                                                                                                                                                                                                                                                      `atatGATCGTTTCTGACATCGAAGCTAACGCCCTCTTAGAGAGCGTCACTAAGTTCCACTGCGGGGTTATCTACGACTACTCCACCGCTGAGTACGTAAGCTACCGTCCGAGTGACTTCGGTGCGTATCTGGATGCGCTGGAAGCCGAGGTTGCACGAGGCGGTCTTATTGTGTTCCACAACGGTCACAAGTATGACGTTCCTGCATTGACCAAACTGGCAAAGTTGCAATTGAACCGAGAGTTCCACCTTCCTCGTGAGAACTGTATTGACACCCTTGTGTTGTCACGTTTGattcattc       <  1:585074/301‑1 (MQ=255)`  
                                                                                                                                                                                                                                                                                                          `gATCGTTTCTGACATCGAAGCTAACGACCTCTTAGAGAGCGTCACTAAGTTCCACTGCGGGGTTATCTACGACTACTCCACCGCTGAGTACGTAAGCTACCGTCCGAGTGACTTCGGTGCGTATCTGGATGCGCTGGAAGCCGAGGTTGCACGAGGCGGTCTTATTGTGTTCCACAACGGTCACAAGTATGACGTTCCTGCATTGACCAAACTGGCAAAGTTGCAATTGAACCGAGAGTTCCACCTTCCTCGTGAGAACTGTATTGACACCCTTGTGTTGTCACGTTTGATTCATTCCAAc   >  1:243732/1‑301 (MQ=255)`  
                                                                                                                                                                                                                                                                                                           `aTCGTTTCTGACATCGAAGCTAACGCCCTCTTAGGGAGCGTCACTAAGTTCCACTGCGGGGTTATCTACGACTACTCCACCGCTGAGTACGTAAGCTACCGTCCGAGTGACTTCGGTGCGTATCTGGATGCGCTGGAAGCCGAGGTTGCACGAGGCGGTCTTATTGTGTTCCACAACGGTCACAAGTATGACGTTCCTGCATTGACCAAACTGGTAAAGTTGCAATTGAACCGAGAGTTCCACCTTCCTCGTGAGAACTGTATTGACACCCTTGTGTTGTCACGTTTGATTCATTCCAAcc  <  1:749757/301‑1 (MQ=255)`  

                                                                                                                                                                                                                                                                                                           `|`                                                                                                                                                                                                                                                                                                              
`GACAGACTGGCTGGAAGAAACCAGAAGTTACCAAGCTGAGTCATAAGGTGTTCGATAAGGACACTATGACCCACATCAAGGCTGGTGATTGGGTTAAGGTTGACATGGGAGTTGTTGGTGGATACGGCTACGTCCGCTCAGTTAGTGGCAAATATGCACAAGTGTCATACATCACAGGTGTTACTCCACGCGGTGCAATCGTTGCCGATAAGACCAACATGATTCACACAGGTTTCTTGACAGTTGTTTCATATGAAGAGATTGTTAAGTCACGATAATCAATAGGAGAAATCAATATGATCGTTTCTGACATCGAAGCTAACGCCCTCTTAGAGAGCGTCACTAAGTTCCACTGCGGGGTTATCTACGACTACTCCACCGCTGAGTACGTAAGCTACCGTCCGAGTGACTTCGGTGCGTATCTGGATGCGCTGGAAGCCGAGGTTGCACGAGGCGGTCTTATTGTGTTCCACAACGGTCACAAGTATGACGTTCCTGCATTGACCAAACTGGCAAAGTTGCAATTGAACCGAGAGTTCCACCTTCCTCGTGAGAACTGTATTGACACCCTTGTGTTGTCACGTTTGATTCATTCCAACC  >  NC_001604.1/14057‑14656`

  

Alignment Legend

Aligned base mismatch/match (shaded by quality score): `ATCG/ATCG` `< 3 ≤` `ATCG/ATCG` `< 12 ≤` `ATCG/ATCG` `< 27 ≤` `ATCG/ATCG` `< 37 ≤` `ATCG/ATCG` `< 38 ≤` `ATCG/ATCG`

Unaligned base: `atcg`    Masked matching base: `atcg`    Alignment gap: `‑`    Deleted base: `‑`

Reads not counted as support for junction

read\_name Not counted due to insufficient overlap past the breakpoint.

read\_name Not counted due to not crossing MOB target site duplication.
